# A safe and simple exposure and Pringle maneuver in laparoscopic anatomical liver resection of segment 7

**DOI:** 10.1186/s12876-023-03056-z

**Published:** 2023-11-29

**Authors:** YongKun Li, Ke Wu, Jing Li, Lu Zheng, Nan You

**Affiliations:** Department of Hepatobiliary Surgery, The Second Affiliated Hospital, Third Military Medical University (Army Medical University), Chongqing, 400037 China

**Keywords:** Laparoscopic anatomical liver resection, Segment 7, Exposure, Pringle maneuver

## Abstract

**Background:**

Laparoscopic access to liver segment 7 (S7) is difficult for deep surgical situations and bleeding control. Herein, our proposed laparoscopic technique for S7 lesions using a self-designed tube method is introduced.

**Methods:**

Clinical data of patients who underwent laparoscopic anatomical liver resection of S7 (LALR-S7) with the help of our self-designed tube to improve the exposure of S7 and bleeding control in the Second Affiliated Hospital, Third Military Medical University (Army Medical University) from April 2019 to December 2021 were retrospectively analyzed to evaluate feasibility and safety.

**Results:**

Nineteen patients were retrospectively reviewed. The mean age was 51.3 ± 10.3 years; mean operation time, 194.5 ± 22.7 min; median blood loss, 160.0 ml (150.0–205.0 ml); and median length of hospital stay, 8.0 days (7.0–9.0 days). There was no case conversion to open surgery. Postoperative pathology revealed all cases of hepatocellular carcinoma (HCC). Free surgical margins were achieved in all patients. No major postoperative complications were observed. Patients with postoperative complications recovered after conservative treatment. During outpatient follow-up examination, no other abnormality was presented. All patients survived without tumor recurrence.

**Conclusions:**

The preliminary clinical effect of our method was safe, reproducible and effective for LALR-S7. Further research is needed due to some limitations of this study.

**Supplementary Information:**

The online version contains supplementary material available at 10.1186/s12876-023-03056-z.

## Introduction

Since the first laparoscopic liver resection (LLR) was reported in the early 1990s [1], the use of laparoscopy for liver surgery has increased rapidly [2, 3]. Proper exposure of the operative field and access to surgical instruments are the basis for safe liver surgery. However, laparoscopic anatomical liver resection of segment 7 (S7) (LALR-S7) is classified as one of the most difficult procedures to perform because of the tumor location due to poor accessibility, rendering exposure of a proper operative field, handling of instruments difficult and poor control of bleeding during the operation even for an experienced surgeon [4]. Various solutions for visualization, such as the hanging maneuver, hand-assisted technique, rotating or elevating device and transthoracic approach, and for hemorrhage, such as laparoscopic extracorporeal or intracorporeal Pringle maneuver, have been suggested to overcome the difficult task, but this problem has not been completely resolved thus far [5–7]. During laparoscopic anatomical liver resection of S7 (LALR-S7), a suitable solution for visualization and hemorrhage should be best to integrate, performed not only to minimize bleeding during parenchymal transaction but also to provide a better surgical field and increased maneuverability in the cramped operative space. Therefore, we describe a surgical technique using a self-designed tube to perform these two activities (exposing lesions in S7 and bleeding control during liver resection) simultaneously, aiming to obtain a clear operative view and easier access to perform LALR-S7.

## Patient and methods

### Patients

From April 2019 to December 2021, patients who underwent LALR-S7 with a self-designed tube to improve the exposure of S7 and bleeding control and met the inclusion and exclusion criteria at our hospital were retrospectively enrolled. The inclusion criteria in this study were as follows: (1) male or female patients aged between 18 and 75 years old, (2) patients who underwent LALR-S7 with the help of our self-designed tube, (3) histologically confirmed HCC and (4) Child–Pugh class A or B liver function. The following exclusion criteria were applied: (1) patients with severe organ dysfunction and (2) patients who underwent laparoscopic anatomical liver resection of S7 with a self-designed tube combined with the resection of other organs except for cholecystectomy. Our institution instituted a formal multidisciplinary tumor board for the treatment of HCC. All new HCC cases were presented for decision making and discussion. Hepatitis B virus (HBV) patients received the whole course of antiviral treatment. Prophylactic antibiotic therapy was administered to all patients 30 min before the surgery and maintained until the second postoperative day. All patients were treated with a full course of postoperative protection of liver function, hemostasis, analgesia, rehydration and other symptomatic and supportive care. Informed consent was obtained according to the Declaration of Helsinki and current ethical guidelines, and written informed consent was obtained from individual participants included in the study. The study was approved by the Ethics Committee of the Second Affiliated Hospital of Third Military Medical University (Army Medical University).

### Methods

**Fig. 1:**
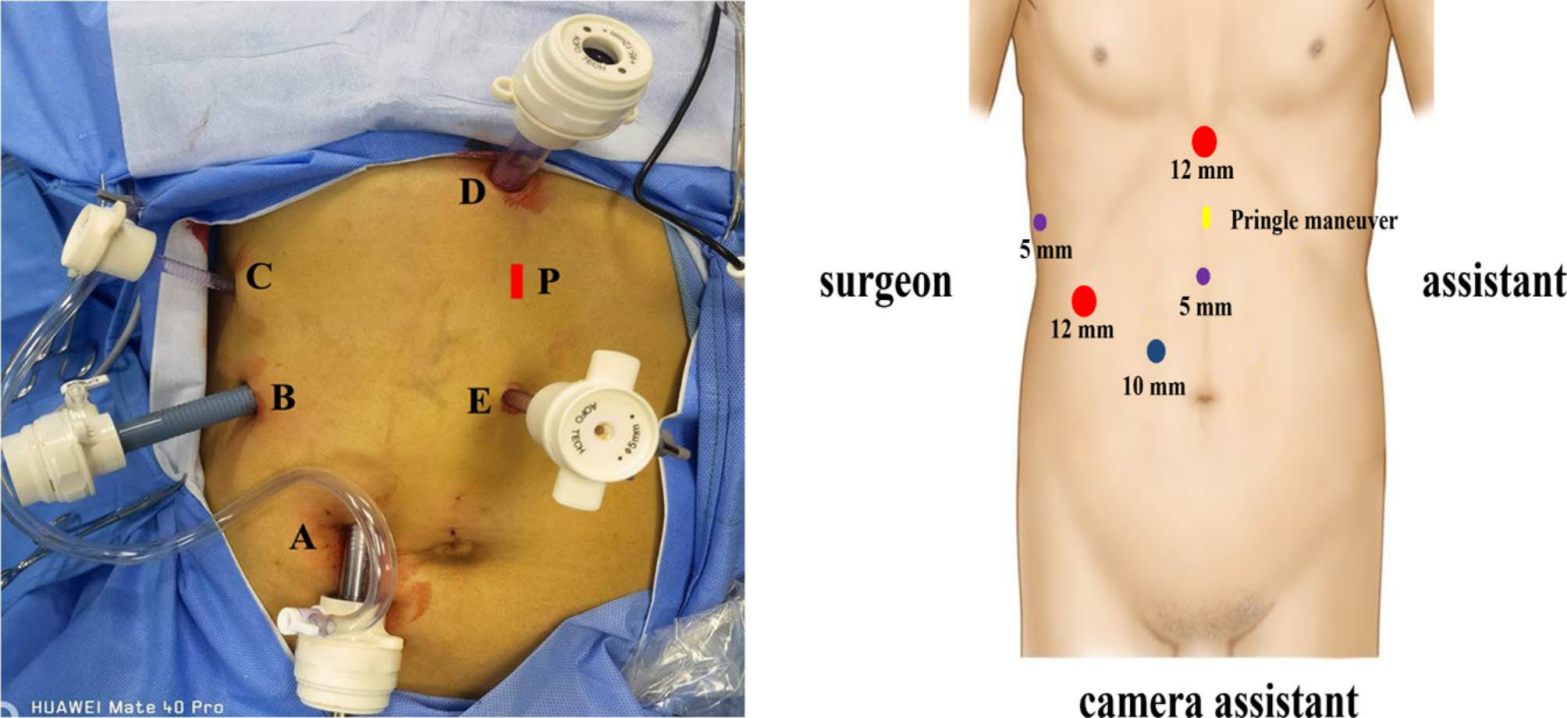
Diagrams of trocar placement for LALR-S7. Two 12-mm trocars, two 5-mm trocars and one 10-mm trocar are used. A: A 10 mm trocar was placed approximately 2.5 cm to the upper right of the umbilicus for scope. B: A 12 mm trocar was placed at the intersection of the right anterior axillary line and 3 cm below the costal margin. C: A 5 mm trocar was arranged through the superior edge of the 11–12th costal bone on the right postaxillary line. D: A 12 mm trocar was placed at the subxiphoid. E: A 5 mm trocar was placed approximately 6 cm below the xiphoid process. P: 3 mm incision for insertion of extracorporeal Pringle maneuver

**Fig. 2:**
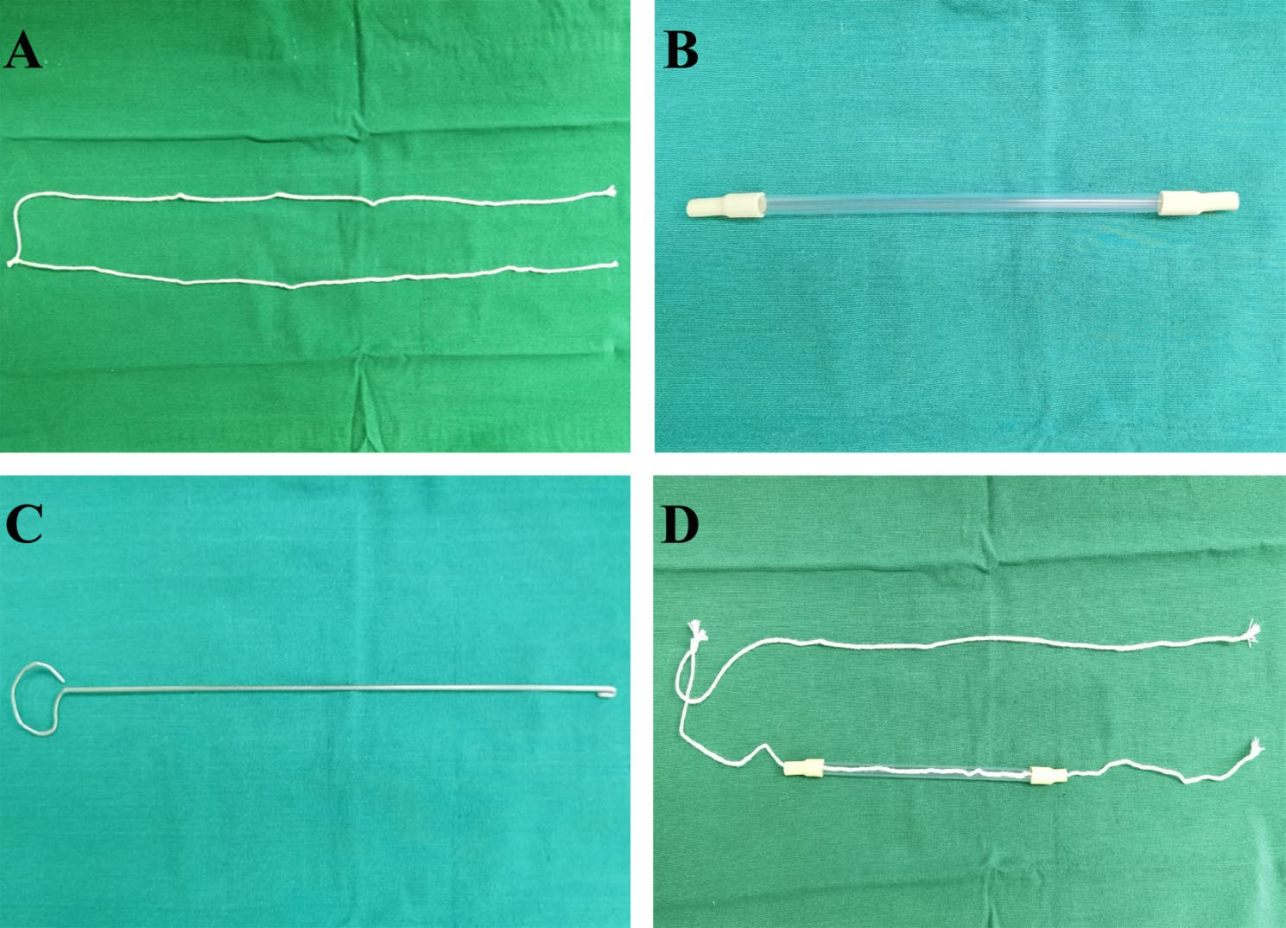
An illustration and image of the laparoscopic first hepatic hilum blood flow occlusion device. (**A**) First hepatic hilum occlusion wire. (**B**) Supporting tube connected with the rubber pipes. (**C**) Leading-out device. (**D**) The laparoscopic first hepatic hilum blood flow occlusion device is completed

**Fig. 3:**
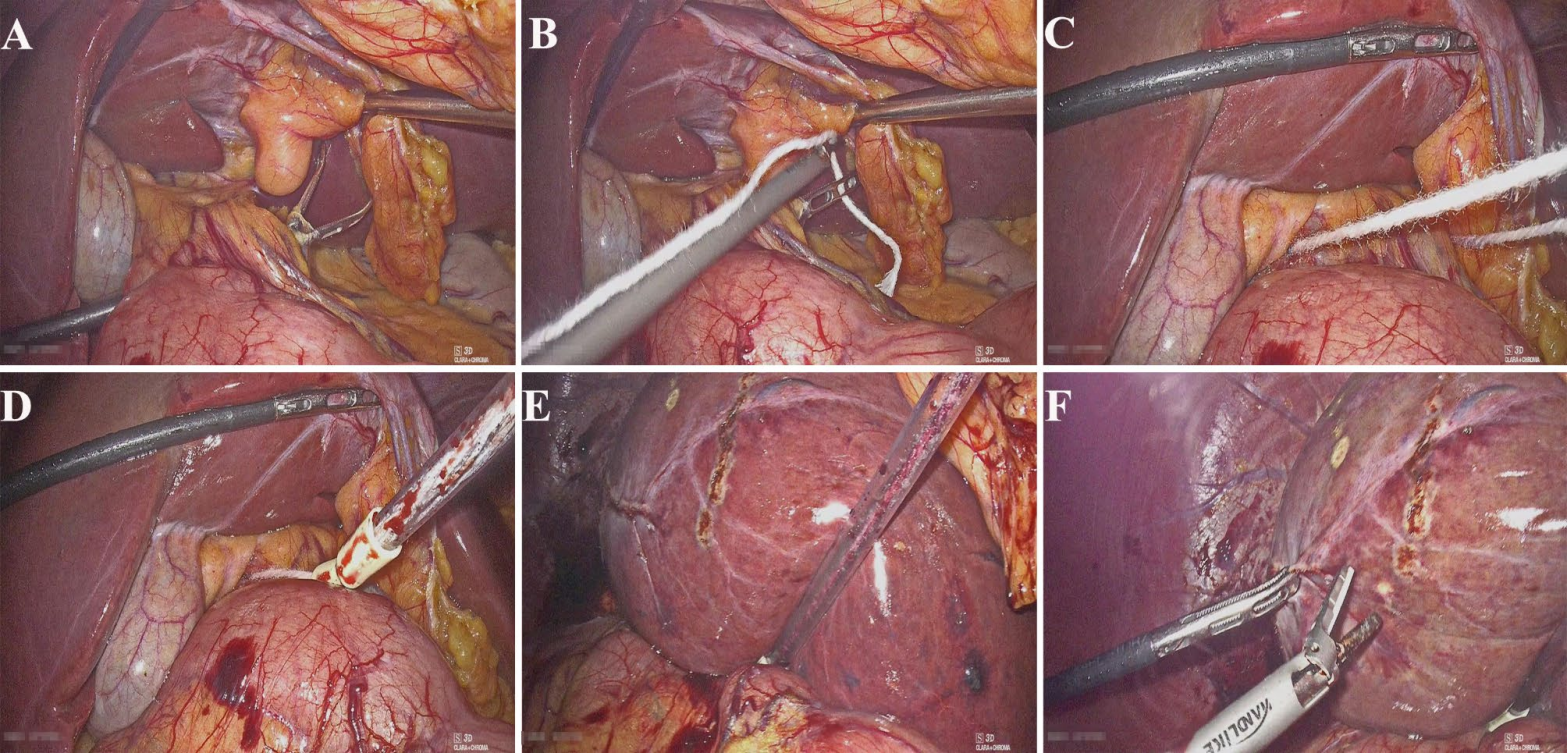
First, a hepatic hilum blood flow occlusion device was used in LALR-S7 to expose lesions in S7 and control bleeding. (**A**, **B**) Small epiploon opening. Forceps are inserted perpendicular to the hepatic pedicle. (**C**) The cord is inserted to surround the hepatic pedicle. (**D**) The cord is exited, and a tube is placed to perform the clampage. The surrounding hepatic pedicle can be clamped easily at any moment by pulling the cord through the tube. (**E**, **F**) The liver was held down using the supporting tube to secure the surgical field

The surgical techniques for laparoscopic liver resection used at our center have been previously described [8, 9]. Under general anesthesia, patients were placed in the supine position in the reverse Trendelenburg position with head up 30° and leg splitting. The monitor was located on the left front of the patient, while the surgeon stood on the right side of the patient, the camera assistant stood between the spread legs, and the assistant stood on the left side of the patient. Pneumoperitoneum was established and maintained with CO_2_ at 12–14 mmHg. Low central venous pressure (lower than 5 cmH_2_O) was maintained by anesthesia to control blood loss. Five trocars were typically inserted in a fan shape around the operation area. A 3-mm length incision was made between the left two ports to prepare for extracorporeal Pringle’s maneuver (Fig. 1). The lesion located in S7 was invisible using the conventional laparoscopic approach. Therefore, the most important aspect of LALR-S7 was to render S7 accessible. The operation began with division of the liver ligaments and right liver mobilization. To better rotate the right liver, we mobilized the right liver from the diaphragm sufficiently, especially the space and the second hepatic hilum to obtain the operative field. During mobilization, the assistant held both sides of the gauze strip with both hands to form a semiclosed traction belt, to retract the liver medially, and posterior ligament dissection was performed with the right hand using a harmonic scalpel through the right subsidiary port (video 1). We developed a self-designed laparoscopic first hepatic hilum occlusion device for laparoscopic hepatectomy to conveniently, rapidly and effectively occlude the first hepatic hilum blood flow to control bleeding. The laparoscopic first hepatic hilum blood flow occlusion device consists of a plastic supporting tube (made of a disposable plastic aspirator, approximately 25 cm long), a first hepatic hilum occlusion wire (a cotton tape, approximately 90 cm long) and rubber pipes made of Fr16 T tube drainage (Fig. 2). The components were readily available in the operating room. The occlusion device was preset before parenchymal transection. During the operation, once the hepatoduodenal ligament had been exposed, the pars flaccida of the gastrohepatic ligament was opened using hook electrocautery. The left-hand grasper was passed through the foramen of Winslow without any instrument guidance. The tip of the hepatic hilum blood occlusion wire was held by the left-hand grasper and first passed through the omental foramen, pulled to the halfway point with two laparoscopic graspers, and both ends were brought out extracorporeally via the 3-mm length incision made between the left two ports. The position of the 3-mm length incision could be adjusted according to the morphology of the patient. Then, two ends of the plastic supporting tube were connected with rubber pipes of approximately 2 cm to protect the first hepatic hilum and the closed pneumoperitoneum. Guided by the occlusion wire, the supporting tube connected with rubber pipes was placed into the abdominal cavity through a 3-mm incision. Our policy was to use intermittent clamping with 15 min (normal liver) or 10 min (chronic liver disease or chemotherapy-induced liver injury) of clamping and 5 min of unclamping. Compared with the traditional method, an extracorporeal laparoscopic first hepatic hilum blood flow occlusion device could make the operation more convenient and safe and cause less damage. After being mobilized sufficiently and pushed, the right liver begins to rotate and rise from the deep and lateral subphrenic rib cage. When the first hepatic hilum blood flow occlusion was needed, under direct vision, the supporting tube connected with the rubber pipes was guided to the first hepatic hilum through the ends of the occlusion wire to block the first hepatic hilum blood flow and the internal end to stay against the pedicle and the external end tightened with mosquito forceps to remain outside of the patient. Liver reperfusion was achieved by removing the mosquito forceps and the supporting tube connected with the rubber pipes was then released to restore hepatic inflow. Then, the liver was held down using the supporting tube to turn the lesion behind the liver dome forward and secure the surgical field, thus expanding surgical vision and providing straight access (Fig. 3 and video 2).

After excellent S7 exposure was achieved, the hepatic pedicle supplying S7 was dissected to find and ligate to obtain the ischemic line, which determined the boundary between segment 6 and S7. Then, S7 was retracted medially by our self-designed tube, and liver parenchymal transection along the ischemic line led to the trunk of the right hepatic vein (RHV). The RHV was then dissected to its root, after ligating and cutting all the branches of the RHV draining the S7. Bleeding of the section was fully stopped, and the section was carefully examined for active bleeding or bile leakage. The resected specimen was inserted in a disposable bag and extracted from an appropriately enlarged puncture hole. A drainage catheter was positioned in the liver section and brought out through the puncture hole, and the abdominal wall incision was then closed layer by layer.

### Statistical analysis

Suitable descriptive statistics were used for different variables. Age, operation time, tumor-free margin and follow-up time are expressed as the mean ± standard deviation and blood loss and postoperative hospital stay are presented as the median and interquartile range. All calculations were performed using SPSS version 22.0 (IBM SPSS, Inc, Chicago, IL) statistical software.

## Results

A total of 19 patients (mean age 51.3 ± 10.3 years) were included in this study. Preoperative evaluations, including blood biochemistry and tumor marker analyses, imaging examinations, indocyanine green clearance tests, and 3-dimensional reconstruction, were routinely performed. Postoperative pathological analysis revealed that all patients had HCC. No patient was converted to open surgery. The mean operation time was 194.5 ± 22.7 min, the median blood loss was 160.0 ml (150.0–205.0 ml), and no blood transfusion was performed during the operation. All the tumor resection margins were histopathologically negative and the mean tumor-free margin was 10.6 ± 1.3 mm. The median postoperative hospital stay was 8.0 days (7.0–9.0 days). There were no cases of mortality. According to the Clavien Dindo classification, postoperative complications included Grade I complications (pleural effusion, n = 3; liver section effusion, n = 2) and Grade II complications (pneumonia, n = 3). No Grade III or above complications occurred. All complications were successfully treated by conservative treatment.

The mean follow-up time was 19.3 ± 7.7 months. During the follow-up period, no hemorrhage, bile leakage, or other complications occurred. No reoperation or perioperative mortality occurred during the follow-up period. No tumor recurrence was noted in any patient at follow-up.

Because this cohort in our study was composed of selected patients without avoiding some confounding variables, which may introduce potential selection bias and affect these results, our results should be interpreted with caution. However, our initial results still provide some important insights into the surgical approach for exposure and the Pringle maneuver in LALR-S7.

## Discussion

S7 lies in the deep area of the subphrenic rib cage. The current laparoscopic approach usually uses linear devices. When performing LALR-S7, we must rely on the hepatic plasticity of patients to a certain degree and obtain a stable surgical view for straightforward access to the operative target, but it is difficult to achieve and maintain using conventional laparoscopic surgery [10]. Some surgeons used the suture hanging maneuver to perform LALR-S7 to avoid poor surgical field exposure, but the technical requirements for suturing and knotting are relatively high and the hanging stitches can cause hepatic laceration by inappropriate pulling [11]. The transthoracic approach is employed for optimum exposure during LALR-S7, however, the critical problems of this procedure are that most surgeons have not got used to perform anatomic resections from the top, and it has increased thoracic organ injuries and postoperative pneumothorax using intercostal trocars [12]. Some surgeons employed a hand-assisted technique to facilitate liver exposure, however, the biggest concern of the hand-assisted technique would be that a larger incision, air leakage and fatigue in the inserted hand, and some surgeons consider that this approach is not suitable for lesions in S7 and S8 [13]. The rotating or elevating device approach is safe and effective for laparoscopic exposure, however, this approach is technically challenging and requires special devices, such as bags, service pipes and pressure infusion systems, and some centers do not have these conditions [5]. Robotic liver resection is also considered a potential surgical approach for S7 resection because it utilizes articulating instruments, which enables performing S7 resection with fewer surgical procedure limitations. However, it also has some disadvantages, such as a lack of flexibility of the camera system and loss of tactile hepatic feedback during tissue manipulation. Robotic liver resection of S7 has not been sufficiently studied, and further investigations are needed to assess its safety and efficacy [14]. Another difficulty of LALR-S7 is prone to bleeding when the hepatic parenchyma is transected. S7 is the drainage area of the RHV, which is usually the main draining venous of the liver, with a large diameter and many branches. In the supine position, among the 3 main veins, the horizontal location of the RHV is the lowest, and most are even below the plane of the retrohepatic inferior vena cava. Meanwhile, the RHV would greatly deviate from the inherent anatomical position during live mobilization. According to hydrodynamic principles, venous return is likely to be obstructed, causing the highest venous pressure. The RHV should be exposed and used as a landmark to guide the hepatic transection plane when LALR-S7. When the liver is transected and the RHV is exposed, the cutting surface and small branches of the hepatic veins are prone to bleeding [15]. As the amount of intraoperative blood loss has previously been reported to correlate with postoperative surgical site infections, intraoperative blood loss is an important factor that considerably affects the surgical outcome of hepatic resection, mainly in terms of the occurrence of postoperative complications. The Pringle maneuver remains the most evidence-based type of inflow occlusion. When applied during LLR, the Pringle maneuver is a quick and safe method of gaining control for inflow occlusion and facilitates low conversion rates. We routinely performed the Pringle maneuver, but liver injury and postoperative portal vein thrombosis due to intimal damage caused by crushing injury of the hepatoduodenal ligament seldom occurred. When needed, the exposure and bleeding control method should be simple, quickly installable, reproducible, cost-effective, applied simultaneously and more importantly, efficient and should not interfere with the surgeon’s actions [16]. We successfully utilized a novel device that provided a good view of the laparoscopic camera and straight access to S7, together with easy control of bleeding, which made LALR-S7 easier.

Its characteristics include the following: (1) The hepatic hilum blood occlusion wire is flexible, and the rubber pipes have toughness and elasticity. When compressing the hepatic hilum, it can block the blood flow into the liver without damaging the soft tissue of the hepatic hilum. (2) The hepatic hilum blood occlusion wire, plastic supporting tube and rubber pipes are all disposable, which meets the requirements for infection control in hospitals. In particular, they are simple and convenient, and can be carried out within a short amount of time. In addition, they are readily available and inexpensive, hardly increasing the economic burden on patients. (3) Some surgeons have found that some specific instruments may be needed for encircling the hepatoduodenal ligament to overcome the blind deployment of tape between the pedicle and the vena cava. However, the use of our method does not require any specific instruments or advanced laparoscopic techniques. The right 12-mm port is a more easy approach for grasper passed behind the hepatoduodenal ligament without injury to the portal vein or vena cava. (4) Our method requires an extra 3-mm incision. However, after the specimen was resected, the incision for extracorporeal Pringle maneuver was extended to the incision for the left subsidiary port for removal of the specimen. Therefore, it does not increase the trauma of patients. (5) The laparoscopic extracorporeal Pringle maneuver combines all the well-known advantages of the technique used in open surgery and appears to carry more practical advantages than the intracorporeal technique. This method blocks the hepatic hilum blood flow outside the abdominal cavity, with a simple operation. By doing so, it can rapidly and repeatedly occlude the hepatic hilum without any restrictions, improve the identification of hepatic veins and Glissonian pedicles in a bloodless/almost bloodless field, reduce the intensity of the bleeding, fully evaluate the bleeding source and importantly, easily control bleeding (if any) to obviate conversion to open surgery. This would help reduce the stress experienced by the operator while keeping the surgical field of vision dry. (6) This method can be used not only for traction and exposure but also to free assistants from retracting the liver and enables them to assist other surgical procedures, which is convenient for surgical operations. Compared to those reported in some of the available LALR-S7 references, this method may be associated with fewer operation times, less blood loss, fewer blood transfusions, shorter hospital stays, and fewer postoperative complications.

The goal of laparoscopic anatomical hepatectomy is to simplify the complicated surgery and design a reasonable, effective and safe exposure mode. Our method seems to reduce the difficulty of LALR-S7 and is easy to popularize and apply, but there are still many problems worth further discussion: (1) The safety of this technique still needs to be verified by a prospective, multicenter, large-sample, randomized, controlled study. (2) Avoiding tumor crush has been one of the most important principles of oncological surgery and was developed to prevent seeding and tumor cell dissemination. No patients had tumors crushed in this study. Therefore, our method may not only prevent putting extreme pressure on focal areas but also eliminate the risk of crushing the tumor. However, our results should be interpreted with caution because our cohort was composed of selected HCC patients (HCC < 5 cm) without comparing the safety and results of the operations with and without crushing, which is needed for more accurate evaluation. Moreover, our method requires no intraoperative blood transfusion and negative surgical margins, which may have a long-term survival benefit for patients with HCC. However, it is still controversial whether this technique can benefit the long-term survival of malignant tumors of the liver, and further research is needed. (3) If the patient had received hepatobiliary surgery, it would be more difficult to pass the tape through the foramen of Winslow due to adhesion. However, such a limitation occurs similar to other methods of the intracorporeal Pringle maneuver. (4) For patients with liver cirrhosis or severe fatty tissue, the absence of flexibility in the liver could make it more difficult to expose by our method, which may lead to a prolonged operation time and postoperative liver damage.

## Conclusion

**Fig. 4:**
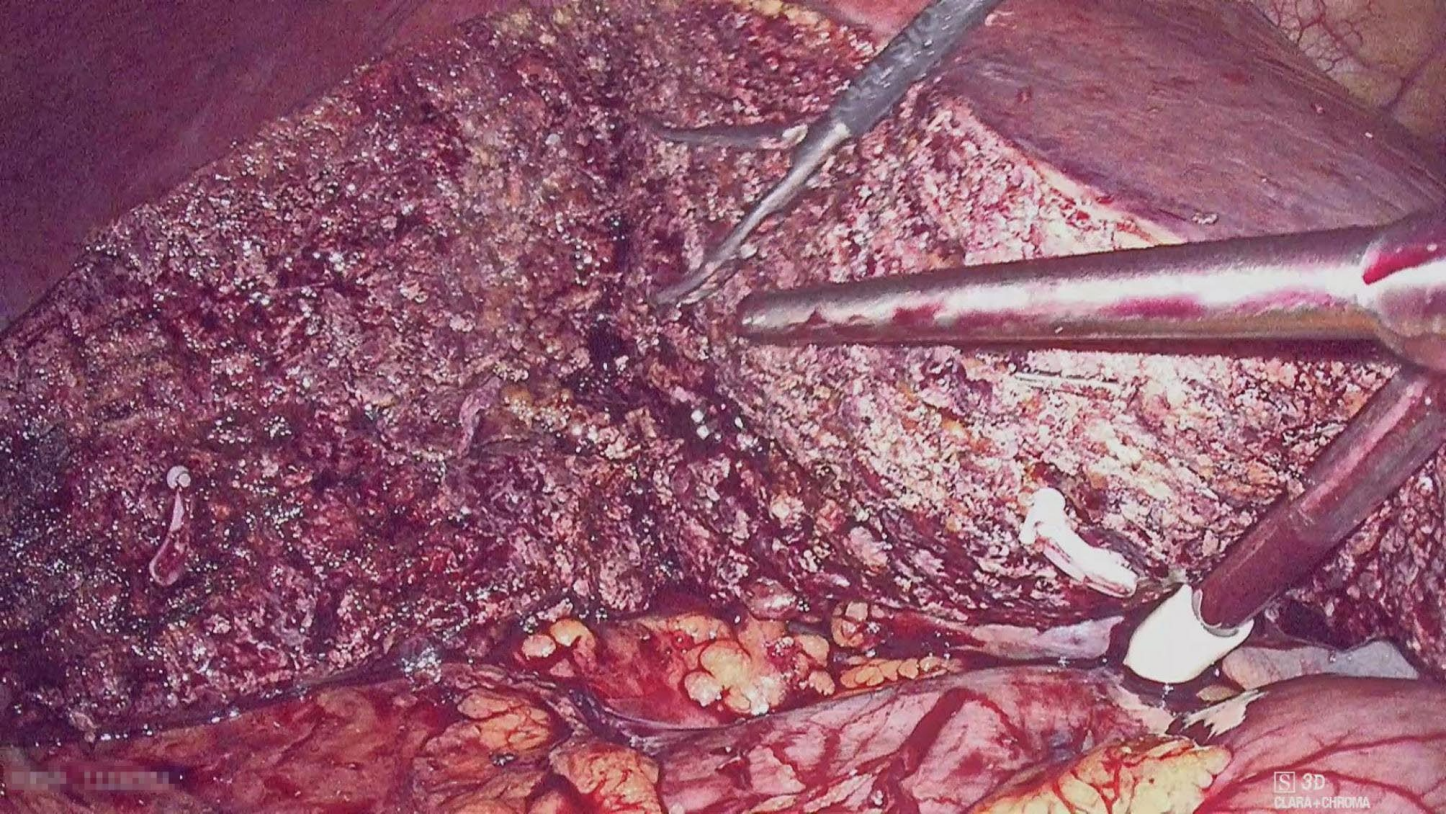
First hepatic hilum blood flow occlusion device used in segment 6 or right posterior lobe hepatectomy

In conclusion, this study assessed the feasibility and outcomes of exposure and the Pringle maneuver in LALR-S7. Our method is safe, reproducible and effective for lesions located in S7. It is not only handy and accessible to acquire but also an effective way to laparoscopically expose and control bleeding during surgery. It can expand the indication for lesions located in segment 6 or right posterior lobe hepatectomy (Fig. 4), however, the advantages of this method are more obvious in S7. However, given the small number of cases, clinical studies with a high level of evidence are still needed to confirm its superiority to other surgical methods. Therefore, the endoscopic surgical method of exposing lesions in S7 and bleeding control during liver resection requires further study.

### Electronic supplementary material

Below is the link to the electronic supplementary material.

Supplementary Material 1

Supplementary Material 2

## Data Availability

The raw data of the current study are not publicly available due to the protection of participants’ personal information but are available from the corresponding author on reasonable request.

## Abbreviations

HBV: Hepatitis B virus
HCC: Hepatocellular carcinoma
LALR-S7: Laparoscopic anatomical liver resection of S7
LLR: Laparoscopic liver resection
RHV: Right hepatic vein
S7: Segment 7
